# Tumor vasculogenic mimicry formation as an unfavorable prognostic indicator in patients with breast cancer

**DOI:** 10.18632/oncotarget.16919

**Published:** 2017-04-07

**Authors:** Yanwei Shen, Jianfeng Quan, Mengying Wang, Shuting Li, Jiao Yang, Meng Lv, Zheling Chen, Lingxiao Zhang, Xiaoai Zhao, Jin Yang

**Affiliations:** ^1^ Department of Medical Oncology, The First Affiliated Hospital of Xi’an Jiaotong University, Xi’an, Shaanxi, 710061, P.R. China; ^2^ Department of Oncology, The Second Affiliated Hospital of Shaanxi University of Chinese Medicine, Xianyang, Shaanxi, 712000, P.R. China; ^3^ Institute of Endemic Diseases, Xi’an Jiaotong University Health Science Center, Xi’an, Shaanxi, 710061, P.R. China; ^4^ Key Laboratory of Environment and Genes Related to Diseases, Xi’an Jiaotong University Health Science Center, Xi’an, Shaanxi, 710061, P.R. China

**Keywords:** vasculogenic mimicry, breast cancer, prognosis

## Abstract

Vasculogenic mimicry (VM), a newly defined pattern of tumor blood perfusion, describes the functional plasticity of aggressive tumor cells forming de novo vascular networks and is associated with the cancer progression and metastasis. However, the VM-positive rate and the impact of VM status on breast cancer patients’ clinicopathological parameters and prognosis remain unclear. Thus, we performed a meta-analysis by incorporating all available evidence to clarify these issues. Eight studies that involved 1,238 breast cancer patients were eligible for inclusion in our study. We found the VM-positive rate was 24% (pooled proportion was 0.24, 95% CI= 0.13–0.34), and VM was significantly associated with larger tumor size (>2 cm) (OR=0.49, 95% CI=0.26-0.90, *P*=0.02) and lymph node metastasis (OR=0.27, 95% CI=0.13-0.57, *P*=0.0005). A boardline correlation was also identified between VM and poorer differentiation (Grade II-III) (OR=0.07, 95% CI=0.00-1.24, *P*=0.07). Nevertheless, no statistically significant associations were observed between VM and hormone receptor and human epidermal growth factor receptor 2 status. Moreover, the results showed that breast cancer patients with VM-positive have a shorter overall survival than those with VM-negative (HR=0.23, 95% CI=0.08-0.38,*P*=0.003). In summary, VM was associated with more aggressive tumor phenotype and poor prognosis in patients with breast cancer. Developing strategies against the VM formation would be a promising therapeutic approach to breast cancer.

## INTRODUCTION

Breast cancer remains the most frequently diagnosed malignant tumor among women and a leading cause of cancer death in females worldwide [1, 2]. Despite the more comprehensive understanding of the breast cancer biology and the development of new protocols for individual treatments, the recurrence and distant metastasis remains an insurmountable challenge. Even when breast cancer tissues have the same clinicopathologic parameters and hormone receptor statuses, tumors can still have different biologic behaviors, including invasive abilities and metastatic potentials. Although the molecular mechanisms have not been elucidated completely, it is well established that the vascular network formation may play crucial roles in these differences [3, 4]. This process supplies blood for tumor growth, invasion, dissemination and metastasis, which have long been regarded as hallmark of tumorigenesis [5]. Former researches considered that the endothelial cells-lined vascular networks were the unique way for tumor perfusion [6, 7]. Thus, efforts to reduce the growth and spread of breast cancer focused on targeting angiogenesis. This therapeutic strategy of inhibiting endothelial cells forming the neovasculature to disrupt breast cancer growth seemed theoretically sound, but the results of anti-angiogenesis trials have been disappointing [8–10].

Indeed, angiogenesis is not the only process by which tumors establish their blood supply for survival, growth, and metastasis. Recent studies have indicated that there exists a novel non-angiogenesis dependent pathway named vasculogenic mimicry (VM), a vascular-like channels generated by some highly aggressive tumor cells, which can mimic the embryonic vascular network pattern to ensure adequate nourishment for tumor tissue [11–13]. The formation of these channels is vasculogenic and mimicry because neither preexisting vessels nor true blood vessels formed the channels, but tumor cells merely mimic the function of vessels [14]. Based on the aforementioned features, the wall of the VM channels were positive for periodic acid-Schiff (PAS) staining, while tumor cells lining the external wall were negative for endothelial markers (CD31 or CD34) staining [15].

Tumor VM provides growing tumors with a new mechanism of blood perfusion and a potential dissemination route. Since its initial identification in melanoma tumor [16], the presence of VM has been observed in other malignant tumors, including lung cancer, hepatocellular cancer, gastric cancer, ovarian carcinoma, prostate cancer and breast cancer [17–23]. Moreover, a recent meta-analysis concluded that tumor VM is associated with a poor prognosis in patients with gastric cancer [24]. However, the clinical value of tumor VM in patients with breast cancer remains inconclusive. Besides, the rate of VM positivity on tumor tissues of breast cancer is not clear. To clarify these issues, thus, we performed a meta-analysis by incorporating all available evidence to determine the positive rate of VM and the influence of VM on clinicopathological features and prognosis in patients with breast cancer.

## RESULTS

### Screening results

**Figure 1:**
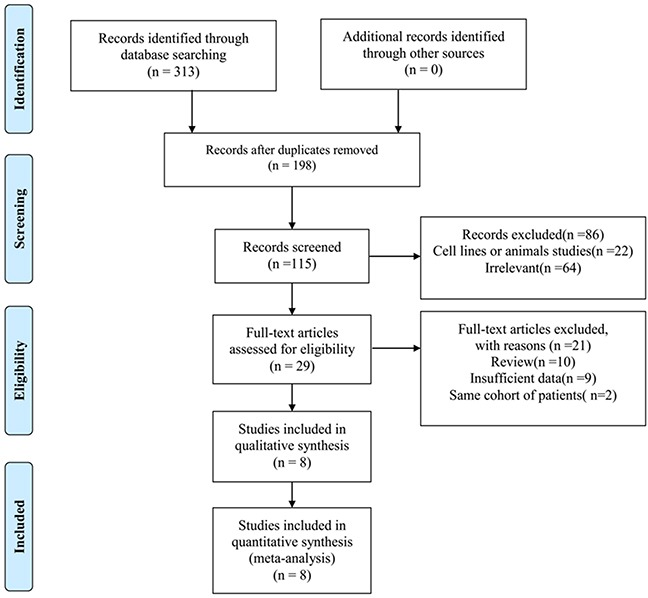
Flow chart depicting the process of selection for this meta-analysis

A detailed diagram of the selection process was shown in Figure 1. Initially, 313 potentially relevant citations were identified through searching of electronic databases as described in the methods. After removing duplicate records, 115 publications were left for screening, of which 86 records were discarded due to studies irrelevant to our aim, or studies without clinical specimens. Sequently, the remaining 29 articles were further evaluated by full-text reviewing, and 21 studies were excluded either owing to being review articles (n=10), lacking necessary information (n=9), being same cohort of patients (n=2). Finally, eight publications [11, 25–31] met all of the inclusion and exclusion criteria, were included in the meta-analysis.

The detailed characteristics of studies are listed in Table 1. In this meta-analysis, the final set of eight eligible studies included a total of 1,238 participants, with individual samples ranging from 80 to 331. Regarding the origin of studies, seven studies were performed in China [11, 26–31] and one [25] was conducted in Japan. All the breast cancer patients were diagnosed by histopathological examination. The VM was determined by PAS staining combined with endothelial markers (CD31 or CD34) staining in seven studies [11, 26–31], while by PAS staining in one study [25]. Additionally, the proportion of patients exhibiting VM formation in individual studies ranged from 5.0% to 49.0%. Eventually, six [11, 25–28, 30] studies reporting the relationship between VM and clinicopathological parameters and three studies [25, 28, 29] concerning the association regarding overall survival (OS) were enrolled. The follow-up period ranged from 60 to 149 months.

**Table 1:** Summary characteristics of the included studies

Study (publication year, country)	Sample size	Recruitment period	Histological type	Methods of VM assay	VM^+^ patients (%)	Clinicopathological parameters	Outcome indexes
Shirakawa et al (2002, Japan)	331	Not available	Mixed	PAS^+^	26(7.9)	Available	OS
Liu et al (2014, China)	90	1998-2005	IDC	PAS^+^CD31^−^	26(28.6)	Available	OS
Zhang et al (2012, China)	146	2006-2010	IDC	PAS^+^CD34^−^	38(26.0)	Available	Not available
Liu et al (2015, China)	91	1997-2005	IDC	PAS^+^CD31^−^	24(26.4)	Not available	Not availabl
Zhang et al (2007, China)	180	2000-2002	IDC	PAS^+^CD34^−^	9(5.0)	Not available	OS
Shen et al (2014, China)	200	2012-2013	Mixed	PAS^+^CD34^−^	98(49.0)	Available	Not available
Liu et al (2013, China)	120	2004-2007	IDC, ILC	PAS^+^CD31^−^	27(22.5)	Available	Not available
Liu et al (2011, China)	80	2006-2009	Unclear	PAS^+^CD31^−^	20(25.0)	Available	Not available

Mixed, ductal carcinoma, lobular carcinoma, medullary carcinoma, scirrhous carcinoma and special; IDC, invasive ductal carcinoma; ILC, invasive lobular carcinoma; PAS, periodic acid–Schiff staining; VM^+^, patients with vasculogenic mimicry formation.

### Study characteristics andquality assessments

Details of the methodological assessment of eligible studies was conducted as shown in Table 2. We evaluated the quality of the seven studies by using the Quality Scale for Biological Prognostic Factors [32]. According to the results of the methodological assessment, the global score of individual studies ranged from 73% to 88%, indicating all the included studies were of acceptable quality.

**Table 2:** Quality assessment of the included studies based on quality scale for biological prognostic factors

Study	Scientific design	Laboratory methodology	Generalizability	Results analysis	Global score (%)
Shirakawa et al	9	11	7	4	78
Liu et al	9	12	8	6	88
Zhang et al	8	12	10	0	75
Liu et al	7	12	8	0	68
Zhang et al	9	12	10	4	88
Shen et al	9	12	10	0	78
Liu et al	9	12	10	0	78
Liu et al	7	12	10	0	73

### Single-arm meta-analysis of VM-positive rate

**Figure 2:**
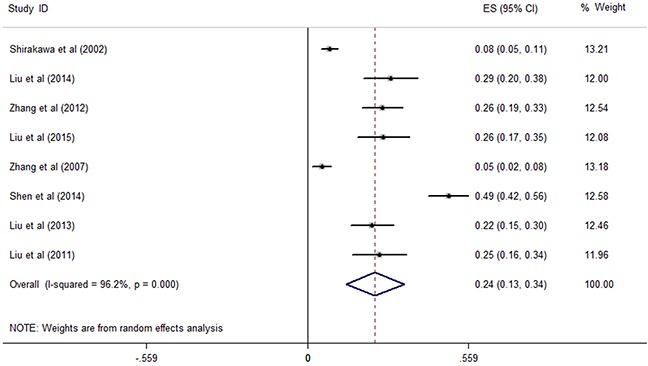
Single-arm meta-analysis of VM-positive rate of on tumor tissues in breast cancer The width of horizontal line represents 95% CI of the individual studies, and the grey boxes represent the weight of each study. The diamond represents the overall summary estimate. The unbroken vertical line was set at the null value (OR=1.0).

The rate of VM-positive in breast cancer was identified using the pooled proportions test method. Based on heterogeneous across the studies, the random-effects model was used for further analyses. The pooled data indicated a proportion value of 0.24 (95% CI=0.13–0.34) from the and random-effects models, suggesting 24% patients exhibiting VM formation in breast cancer (Figure 2).

### Correlations between VM and clinicopathological parameters

**Figure 3:**
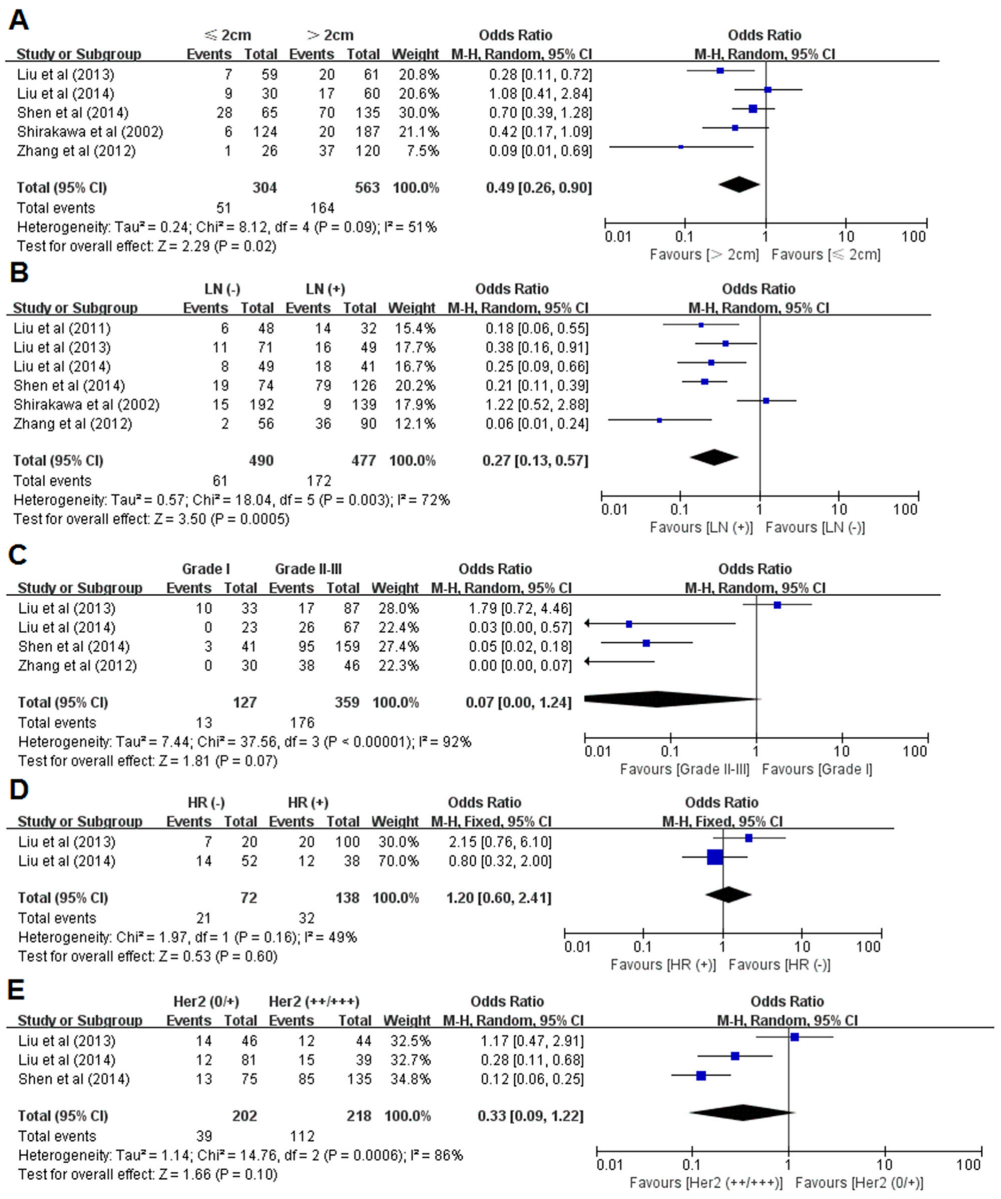
Associations of VM with clinicopathological parameters **(A)** The relationship between VM and tumor size; **(B)** The association between VM and lymph node status; **(C)** The association between VM and histological grade; **(D)** The association between VM and hormone receptor status; **(E)** The association between VM and HER2 status. Abbreviations: LN, lymph node; HR, hormone receptor; M–H, Mantel-Haenszel.

The risk estimate with pooled odds ratios (ORs) were used to determined the associations between the VM and clinicopathological parameters in breast cancer. The meta-analysis results showed that VM was significantly associated with larger tumor size (>2 cm) and lymph node metastasis (OR=0.49, 95% CI=0.26-0.90, *P*=0.02; OR=0.27, 95% CI=0.13-0.57, *P*=0.0005; respectively) (Figure 3A and Figure 3B). Moreover, a boardline correlation was identified between VM and poorer differentiation (Grade II-III) (OR=0.07, 95% CI=0.00-1.24, *P*=0.07; Figure 3C). Nevertheless, no statistically significant associations were observed regarding hormone receptor and human epidermal growth factor receptor 2 (HER2) status (OR=1.20, 95% CI=0.60-0.90-2.41, *P*=0.60; OR=0.33, 95% CI=0.09-1.22, *P*=0.10; respectively) (Figure 3D and Figure 3E).

### Impact of VM on OS

**Figure 4:**
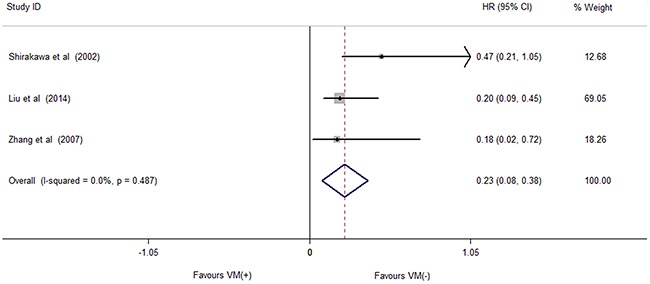
Forest plots of studies evaluating hazard ratios (HRs) of VM for overall survival Breast patients with VM-negative exhibited a longer overall survival than those with VM-positive (HR=0.23, 95% CI=0.08-0.38, *P*=0.003).

The association between the prognostic significance of VM and OS were calculated via meta-analyses of hazard ratio (HRs). No significant heterogeneity was observed (*I^2^*=0.0%, *p*=0.487), thus, the fixed-effect model was used for date analysis. As shown in Figure 4, the pooled estimates demonstrated a significant relationship between VM and shorter OS (HR=0.23, 95% CI=0.08-0.38, *P*=0.003). This indicated thatVM was an adverse prognostic factor in breast cancer.

### Publication bias

Both Begg’s and Egger’s test were conducted to assess the potential publication bias of included studies. The results suggested that there were no obvious publication biases in our meta-analyses (Table 3).

**Table 3:** Begg’s and Egger’s test results for funnel plot asymmetry

Indexes	Begg’s *p* value	Egger’s *p* value
Positive rate of VM	1.00	0.65
Tumor size	0.33	0.25
Lymph node status	0.71	0.43
Histological grade	1.00	0.20
Hormone receptor status	1.00	Cannot be calculated
HER2 status	0.30	0.42
OS	1.00	0.77

## DISCUSSION

For patients with breast cancer, the VM-positive rate and the impact of VM status on patients’ clinicopathological parameters and prognosis remain unclear. A meta-analysis incorporating all available data from related studies is a reasonable approach by which to address these issues. We conducted this study and found nearly 24% of breast cancer patients exhibiting VM formation. Moreover, our pooled results provide compelling evidence of a significant positive association between VM and larger tumor size and lymph node metastasis. Our analysis also indicates that breast cancer patients with VM-positive have a shorter OS than those with VM-negative. Taken together, these results indicated that VM was a promising prognostic indicator for aggressive clinical parameters and worse prognosis.

The underlying mechanisms through which VM might promote breast cancer progression and influence the prognosis are unclear. Several possible scenarios have been proposed. First, VM could provide a functional pathway of perfusion for rapidly growing tumors and possibly a metastatic escape route within the growing tumors that functions either independently of, or simultaneously with, angiogenesis [25, 33]. Second, recent evidence indicates that cancer stem cells are involved in VM formation of breast cancer [22, 23, 31], have been associated with tumor invasion and metastasis. Third, the VM formation of breast cancer involves signaling pathways and some factors related to tumor cell migration and invasion, including Nodal signaling pathway [22], heat shock protein 27 pathway, AURKA protein kinase [23], IL-8 [34] and Claudin-4 [12]. Finally, VM is lined with tumor-derived endothelial-like cells that differ from molecular characterization of conventional tumor angiogenesis. Consequently, VM might present with a native resistance to anti-angiogenic compounds [35].

For every plus, there is a minus. As a meta-analysis, the present study allows us to get a better understanding on the clinical role of VM formation in breast cancer patients by increasing the statistical power through combining data from all available evidence, however, certain limitations in the meta-analysis should drew our attention as well. Firstly, we only included published studies written in English and Chinese, which may cause selection bias. Secondly, although uniform criteria were used in selected eligible studies, inherent differences among studies still existed. Thirdly, the sample sizes of eligible studies with OS data was relatively small, and estimated HRs with corresponding 95% CIs were calculated by Kaplan–Meier curves, which may limited the reliability of results. Finally, all studies included in our meta-analysis were conducted in Asia; hence, the results of this study should be interpreted carefully. Therefore, additional well-designed studies with larger sample sizes and patients of different ethnic backgrounds are highly needed to make a more reliable results. Despite of certain limitations, the present meta-analysis had several strengths. First of all, a substantial number of participants were pooled from different studies, representing a sizeable patient sample and significantly improving on the statistical power of any of the individual analyses included. Then, no publication bias was detected, indicating the reliability of our pooled results.

In conclusion, although certain limitations exist, the results of current study showed that VM was associated with more aggressive tumor phenotype and poor prognosis in patients with breast cancer. These results suggest that developing strategies against the VM formation would be a promising therapeutic approach to breast cancer. Additional well-designed studies with larger and more diverse populations are highly needed to validate our current data.

## MATERIALS AND METHODS

### Search strategy

The meta-analysis was conducted according to the Preferred Reporting Items for Systematic Reviews and Meta-Analyses (PRISMA) statement and guidelines [36]. Electronic databases of Pubmed, Embase, Web of Science and China National Knowledge Infrastructure were comprehensive systematic searched without using language restrictions. The search period was from January 2000 to January 2017. Combinations of the following search string were used to screen for potentially relevant studies: (“vasculogenic mimicry” OR “tumor cell-lined vessels” OR “ tumor derived endothelial cells”) AND (“breast cancer” OR “breast carcinoma”). The bibliographies of all retrieved articles were individually and manually screened to identify additional studies.

### Inclusion and exclusion criteria

To be included in the single-arm meta-analysis of VM-positive rate on tumor tissues, studies had to met all of the following criteria: (1) patients with histologically confirmed breast cancer; (2) VM-positive primary tumor tissues were assessed by PAS staining and/or endothelial markers (CD31 or CD34) staining in the tissue specimens; (3) samples of VM-positive were available. Moreover, studies in the meta-analysis that focused on patients’ clinicopathological characteristics and prognosis according to VM status met the following criteria: (1) studies that examined the relationships between VM and at least one of the following clinicopathological parameters and outcome indexes: tumor size, lymph node status, distant metastasis, histological grade, hormone receptor status, HER2 status and OS; (2) inclusion of sufficient data to estimating the ORs for clinicopathological parameters and HRs for OS. Exclusion criteria were as follows: (1) studies with same cohort of patients reported in other studies; (2) the ORs/HRs and the corresponding 95% CIs unable to be obtained directly or could not be calculated; (3) letters, reviews, case reports or editorials without complete data.

### Data extraction and methodological assessment

The following parameters was independently evaluated and extracted by two investigators (Y.S. and J.Q.) according to a unified standard aforehand proposed: first author, year of publication, study country, research period, sample size, histological type, VM assay methods, VM-positive rate, clinicopathological features and the long-term overall survival. Discrepancies on the eligibility of studies were resolved by full-text review and discussion with a third reviewer (M.W.) until the two original reviewers reached consensus.

The methodological assessment was conducted using the Quality Scale for Biological Prognostic Factors reported previously [32]. Two specialists (J.Y. and J.Q.) who are experienced in clinical and basic experiments independently assessed the quality of each study according to the quality scale (Supplementary File 1). This scale assesses the quality of study based on the following four main aspects: (1) the scientific design; (2) the description of the methods used to identify the VM-positive tumor tissues; (3) the generalizability of research findings; (4) the data analysis of the study. The overall maximum points was 40. The global scores were presented as percentages, ranging 0–100%. Studies with higher proportion values were considered high quality. Discrepancies were discussed with another specialist (M.L).

### Statistical analysis

All statistical analyses were performed by Stata 12.0 software (Stata Corporation, College Station, TX, USA) and Review Manager 5.3 software (Cochrane Collaboration, London, UK). The calculation for VM-positive rate on tumor tissues was conducted using the pooled proportions test. For the summarize of the relationship between VM and clinicopathological parameters, ORs and 95% CI were combined to give the effective value. For the quantitative aggregation of survival results, HRs and their 95% CIs were combined as the effective value. The HRs were calculated from the reported data directly by number of events or calculated from Kaplan–Meier survival curve using Engauge Digitizer version 4.1 software (free downloaded from http://sourceforge.net). The between-study heterogeneity was using Cochran Q and *I*^2^ test. When heterogeneity was not obvious (*I^2^* < 50%, *P*-value for heterogeneity <0.10)[37], a fixed-effect model was applied to pooled data. Otherwise, the random-effect model was used. The significance of the pooled OR or HR was evaluated by Z test and *P*<0.05 was considered significant. The potential publication bias was estimated by Begg’s rank correlation method [38] and the Egger’s weighted regression method [39] (*P*<0.05 considered to be statistically significant).

## SUPPLEMENTARY MATERIALS




