# Supplementary material for: Tumor vasculogenic mimicry formation as an unfavorable prognostic indicator in patients with breast cancer
Source: Oncotarget. 2017 Apr 7;8(34):56408–16. doi: 10.18632/oncotarget.16919 (PMC5593571; doi:10.18632/oncotarget.16919)
Supplement: Supplementary file 1 [file oncotarget-08-56408-s001.pdf]

Tumor vasculogenic mimicry formation as an unfavorable prognostic indicator in patients with breast cancer

SUPPLEMENTARY MATERIALS

Supplementary File 1: Quality scale for biological prognostic factors

See Supplementary File 1
